# New York Odontological Society

**Published:** 1890-11

**Authors:** 

**Affiliations:** New York Odontological Society


### NEW YORK ODONTOLOGICAL SOCIETY.

The New York Odontological Society held its regular monthly meeting Tuesday evening, June 17, 1890, in the New York Academy of Medicine, No. 12 West Thirty-first Street. The Vice-President, Dr. C. A. Woodward, in the chair.

### INCIDENTS OF OFFICE PRACTICE AND CASUAL COMMUNICATIONS.

*Dr. H. C. Meriam.*—Mr. President, I think we are all very much interested in the beautiful forms by which Dr. Perry expresses his ideas in instruments, and I wish to call attention to a very easy way of getting new forms. While it may not be new, I do not know that it is in print. It is to have sent from an instrument-maker untempered instruments about the size desired, spoons, chisels, etc. Then with a brass hammer, on a wooden anvil or block, fashion them into any form, and return them to be tempered and polished. They come so soft that they can be bent as desired, and tried into a cavity. The gentlemen will notice that in the side of these blocks or anvils are holes in which to bend the larger sizes. The object of the brass hammer, which is not original with me, is that any curve may be fashioned without scarring the steel. I will pass these around that all may see how very soft they are, as Mr. Schmidt sends them to me. Here are some instruments of my own forms, and some from English forms given in Ash's catalogues. Some of them may be extra size, but I keep at my work-case a very coarse whetstone, on which I can reduce the size of any instrument.

*Dr. W. H. Dwinelle.*—A young lady presented herself to me about a week ago, with two front teeth very badly discolored, the color corresponding with that of No. 7 of the S. S. White Company's artificial teeth. She wished to have them bleached. Bleaching teeth I have practised many years, and I wrote some articles on the subject in 1848, which were published in the *American Journal of Dental Science.* In this case, as a matter of course, I concluded that the teeth were dead, for they were quite dark; and I, with impunity, opened the pulp-canals. Previously, however, I had armed myself with a few facts in the premises. I learned from her that her age at that time was between eighteen and nineteen, and that the regulating of her teeth began about five years before, which would make a very material difference in this respect; that, provided the pulps had been destroyed at that early age, I must look for an enlarged foramen and pulp-canal, and also for several other things that would accompany that period of life. To my astonishment I found the last third, or possibly half, of the tooth-pulp alive. But I had gone too far to retrace my steps; the only way of getting out was to go through and complete the operation, so I treated the living tissues with carbolic acid. Filling the opening approaching the pulp with carbolic acid, I approached it with a Swedish broach charged with acid, retreating and progressing farther and farther, destroying the nerve tissues as I advanced, until I reached the foramen. With careful management this operation is not painful. There, as I had anticipated, I found a very large foramen,—the tooth a mere shell, with an opening and canal so exceedingly large as to indicate that nutrition had been suspended at an early period, corresponding to thirteen or fourteen years of age; and in fact, although I found the last third of the pulp in the canal comparatively alive, yet it evidently had suspended its function at that period, five or six years before, and to all appearances it was a thirteen-year-old tooth. Yet it had sufficient energy to inject its red blood, charged with iron as you know, into the tubuli of the tooth, discoloring the fibrillæ in the tubuli.

Now, what are you going to do about it, gentlemen? That is what I came to inquire. I have, in conformity with the best authorities, assumed that one great objection to bleaching teeth has been that the bad color, the yellow or black color, would return; so in preparing the tooth I have cleaned out the pulp-canal to the foramen, and have been very particular to hermetically seal it, and now I am treating it with chloroform, in order to dissolve all the fatty substances within the fibrillæ of the tooth. As I have said, the foramen was unusually large, and I found a great deal of blood congested there; not exactly degenerated blood,—that implies a breaking up and degeneration of the blood-corpuscles. I found myself in a multiplicity of complexities. I am now dissolving the fatty substance of the fibrillæ. I have not gone on to the use of chlorine yet, my idea being to get the fluid into the porous texture of the tooth within the tubuli, and get it into a condition to receive the chlorine. I use chlorine water rather than chlorine gas. There I stand in this case, gentlemen. I do not go forward because it seems best to stand still at present. I have probably had larger experience in bleaching teeth, or an experience extending back farther than any one that I know of; I have not heard of any dentist practising it farther back than 1848; but I would like your advice. I consider this is a class meeting, and I want every brother to speak. We know there are a great many reagents for removing discoloration. One of the best in my experience is chlorine. It may be used in various forms, as chloride of sodium and chloride of lime. And a very good reagent also is oxalic acid. It is one of the best reagents against iron that I know of. Oxalic acid is one of the best agents known to remove iron- and ink-stains, as we all know. Sulphuric acid is excellent for bleaching, but it should be used with great caution. I ask your advice in the premises.

*Vice-President Woodward.*—Some of our Executive Committee are now present: I would ask if they have a report to make.

*Dr. Bogue.*—Mr. President, Dr. Lord, the chairman of the Executive Committee, requested me to make the following report:

"The Executive Committee, to whom was referred Dr. Bödecker's proposition to give five thousand dollars under certain conditions for the establishment of a dental club and library, beg leave to report that they recommend the appointment of a special committee to act with committees which should be named by the District Society and the Brooklyn Society to consider this question and present a proper mode of action."

On motion, the report was received.

*Vice-President Woodward.*—If there is nothing further to be presented under Incidents of Office Practice, we will listen to a paper by Dr. Bryan, of Basel, Switzerland, entitled, "Matrix or Ring Fillings," to be read by Dr. Bogue.

(For Dr. Bryan's paper see page 661.)

*Dr. Bogue.*—Mr. President, Dr. Bryan, in a private letter a week or two subsequent to the sending of his paper, called attention to the fact that these matrices which have been passed around the room, can be soldered by the use of a soldering iron, which avoids the possibility of taking the temper out of the steel. The steel is bought in sheets, perhaps an inch wide, and as long as is desired. It is not thicker than very thin paper, and can be cut and shaped with a pair of pocket scissors.

The mandrel spoken of is six or seven inches in length, the lower end not being larger than the reporter's pen-holder, and the upper end being sufficiently large for the largest molar. As has been described in the paper, each half-centimetre is numbered to correspond to the numbers on the little frame which has been passed around. I tried these matrices, at first with indifferent results, but I found a number of cases where they seemed to come handily in, and soon I found myself using them more and more, and the result has been exceedingly gratifying. Where teeth are largely broken down, this thin polished steel matrix can be slipped into place, the filling inserted, and the matrix removed with ease, leaving the filling as smooth and as accurately adapted as has been described, provided always that pains be taken in the insertion of the filling. Of course one understands that the possibility of overlapping the margins is always present and must be looked out for. I consider that the saving of time and labor to the dentist, and worry to the patient, is sufficient to make the matrix worthy of our attention.

*Dr. Howe.*—Mr. President, I will state that French cold-rolled steel, as thin or thinner than the steel of which these matrices are made, can be obtained from Frasse & Co., of No. 92 Park Row, and at other jewellers' supply-stores. When used in a similar manner to that recommended by Dr. Bryan, it serves the purpose admirably.

*Dr. Bogue.*—Mr. President, the driving of a few nails into a block, and numbering them according to the number of the matrix or ring, takes but little time and will amply repay the trouble. In this little box three-fourths of the rings have been used; but if each pin held four or five rings of varying width upon it, it would be but a moment's work to find a matrix that would fit any tooth. A very ordinary workman could make up a hundred of those matrices in a short time.

*Dr. A. H. Brockway.*—Mr. President, this style of matrix, as may readily be seen, is very much like those put out by Dr. Brophy a few years ago, working on the same principle, except that his matrix was retained in place by a set screw rather than by a wedge. I bought some of those matrices when they first came out, but found they were not very easily adjusted, and as they required more trouble than I was willing to give, I have gradually abandoned their use. I have tried nearly all the matrices that have been introduced; some of them with a great deal of satisfaction. The matrix that I have found most serviceable, for use certainly in amalgam fillings alone, is that devised by Dr. Ivory. I presume most of those present are familiar with it. I have found it very easy to adjust, and it serves an excellent purpose, one advantage being that it does not require a space all around the tooth. This one shown here of course does; but the material of which it is made being so thin, that would be a very trifling objection. I am exceedingly well pleased with these, and shall hasten to adopt them. It is surprising to me how little use is made of the matrix by many operators. But a few days since I met a dentist of most excellent standing who told me that he had never used one; and his case is not entirely singular. To me, I must confess, they are indispensable in the saving of time and in enabling me to do better work. This convenient method of arranging them for ready use is very commendable. Little things like that in the way of saving time add so much to one's efficiency. I am always glad to see anything of that kind brought forward, for the reason that I am a great stickler for saving time. Time is the scarcest thing in the world and ought not to be wasted.

*Dr. Dwinelle.*—The matrix is an exceedingly accommodating appliance, adapting itself to varying contingencies. The first matrix I ever saw or used was composed of a plate of rolled gold, bent around between the teeth, not covering or embracing the circumference of the teeth, but wedged to its place. You will find an allusion to it in a treatise on crystalline gold which I wrote about 1854. That is the first intimation that I ever had of the use of the matrix in filling teeth. The ring matrix, however, is the best form in use. If it does not always adapt itself to the entire circumference of the tooth it embraces, it can easily be adapted to its place by slipping a wedge in by the side of the tooth at a point remote from that which is being operated upon, and that will taut it down to place. I think the matrix is one of the most useful and practical instruments that we have in our profession.

*Dr. Bogue.*—It occurred to me when Dr. Brockway said he tried a certain matrix for a little while and then abandoned it, also when I learned that Dr. Dwinelle still persists in the use of chlorine for bleaching teeth when there are lots of other things used for that purpose, that old dogs cannot learn new tricks. I am glad to hear that Dr. Brockway is going to try these matrices. I think every gentleman who will use them will be exceedingly pleased with them.

*Vice-President Woodward.*—Gentlemen, we will now pass to the reading of a paper by Dr. J. Morgan Howe, entitled, "What is the Essential Basis of Professional Ethics, and the Proper Relations of a Profession to a Trade?"

(For Dr. Howe's paper see page 664.)

*Vice-President Woodward.*—Gentlemen, you have listened to the reading of this most excellent paper, and I hope that no time will be lost in beginning the discussion.

*Dr. Brockway.*—I am not in condition to speak at length to-night. Unfortunately, I woke up this morning to find myself almost deaf, through having imprudently ridden in an open car last evening. I have therefore lost much of the paper which has been read, but have heard enough to satisfy me that it is one of the most refreshing and inspiring papers I have ever listened to. I think it must have warmed Dr. Meriam's heart to hear it; and while he objects to being the first one to speak upon it, I am willing to break the ice, and remove his objection, so that we shall presently hear him. I confess to some feeling of shame and contrition while listening to the paper, and appropriated to myself somewhat of the rebuke which was so properly administered to those who had been lacking in their appreciation of the efforts of such men as Dr. Meriam and others, who have been endeavoring to lift up the standard of professional ethics, and to point out the distinction between professional and trade conduct. I think no one who has listened to the paper can fail to be impressed with the unselfishness which has prompted the efforts of these men, and I proffer to them my tribute of sincere respect and admiration for their efforts in this direction.

*Dr. Meriam.*—Mr. President, if my story is below the standard of what is admitted to the dignified proceedings of the New York Odontological Society it can be stricken from the report. I remember attending, when a boy, a church the services of which were held in a building hired from another society which was so impoverished that it was glad to rent it to us, holding their own services at noon-time, which was our intermission. As I came some distance, I often stopped at noon to attend the other service. They had a choir-master who was somewhat officious, and one day, when they had a visiting clergyman in the pulpit, who had just given out a hymn, to my astonishment the choir-master turned to the congregation and said, "We will sing the first, third, and fourth stanzas of the hymn." They sang them through, and then the old minister rose behind his desk, and said, "The congregation will now sing the omitted stanzas of that hymn."

Mr President, we assume that we are assembled together to sing a professional hymn, but in the past some voice has said, "You will please omit verses that discuss certain questions; you will please omit the discussion of materials, and omit the discussion of journalism, instruments, etc., and bring forward only those things which have no direct money value, thus singing the verses that do not annoy any one." But, gentlemen, the time has come, I think, when the profession is going to sing the omitted verses of that hymn.

There was a society in Europe, I believe, for the study of aerial navigation. It had existed for many years, with occasional meetings, at which the members had discussed and reported progress. Of late the results have been more promising, and it has seemed to some of them that success was almost at hand, and that the problem of aerial navigation would soon be solved.

But what has been the effect upon the society? Each member has scented a prospective fortune, and the society has ceased to report and discuss, and has been compelled to disband, in the presence of prospective profit. Now we read that the American Dental Association has run out. We fear there have been some omitted verses that the American Dental Association have not sung. We have consented to the withdrawal of instruments, the composition and formula of filling-materials, etc., as topics of professional study or discussion. This is a question of trade or profession with us; not a question of the name of trade or the name of profession, but as representing two opposite methods. It is impossible, probably, for trade to exist with the freedom that science claims; and it is impossible for science to exist, with its desire to make known, and at the same time submit to, the restrictions of trade. Furthermore, it is particularly impossible for a profession to exist where its members accept service with others whose interests are antagonistic to that profession. In one of the scientific departments of the government, a man was engaged to conduct certain experiments, and at the close of the work he wished to patent the processes and results. This was denied him, as he was hired to conduct certain work, for which he received his remuneration from the government, and thereby lost his title to it. We are bound in honorable contract to our profession on entering it. If a society does not give sufficient return, there is no dishonor in a man stepping out from it; but that he can at the same time claim the right to share in the labors of others and the right to deny to others is to me a contradiction. I am informed that this position has been sustained in a recent decision in one of our courts,—viz., that a man could legally sell beforehand his rights in results when letting himself to conduct experiments, and that under such circumstances he has no claim to the product as his own. Some of the electric companies recognize this in the hiring of their men, the understanding being that everything they shall discover while in the company's employ shall be patented for the company. Now, what business does to protect itself, from business motives, we must do for our profession from professional motives,—discuss and record for it everything relating to its practice.

We wish to render bills for "professional" services, and the courts assume we are bound to render those common to a profession. Thus, both we and the courts assume that our services are the common property of the profession to which we belong, and which all are free to render if possessed of the requisite skill. Now, what makes a patent or secret non-professional is that it is not the profession's, and cannot be used without the consent of others. And our bills might read "For such services as Tom, Dick, or Harry allows us to render."

I hope there will be at Harvard, and other schools, prizes offered for the best thesis on the preparation of some of the filling materials. I do not know why every dental school in the country should not have a pharmacist. I do not know why the attention of men in chemistry should not be called to the chemistry of filling materials. We have such men in our schools, and their teaching, instead of being directed wholly to general chemistry, should be directed somewhat to our specialty, not only to chemistry as it assists in the work of histology and pathology, but also to those things that supply the loss of parts.

In reference to this question of the control, or the attempt to control, our societies, we had a very unfortunate case in Boston. I do not blame any one; the thing has gone on so long that the steps are probably taken without any idea of the reproach they bring on a society. It seems that one of the exhibitors, without asking the consent of the committee, went to one of the electrical companies and had them put in a wire for running his own apparatus, with the understanding that no one else should have the use of it. Now, the advantage of an exhibit before a society is that various inventions or products may be fully shown before them, while it is for the interest of what we may call the trade spirit to keep others out of the way. But I do not think that is consistent with proper pride on the part of our instrument-makers. We, as dentists, must respect good workmen. When dentistry ceases to respect good workmanship, it will lose one of its advantages. But we are not called upon to lose any respect for the workman; we are called on to look at the question of whether a workman shall overstep his bounds and become a director of a society of professional men, and say who shall and who shall not be represented there.

I do not know whether this story reached General Walker, the President of the Institute of Technology, who occupies the highest position in all matters concerning education, but when we went to them again for the use of the Institute of Technology, he made very particular inquiries about the exhibit, what it was to be, and so on, and it ended by my assuming all the responsibility of damage, with the assurance that the exhibit should be educational. The rival exhibits were placed side by side, and in that way we overcame the exclusive idea. I think this, perhaps, gave offence because misunderstood.

I am of course aware of the changed conditions of life brought about by our modern advantages, and that now, in mercantile affairs, some of the finest brains of our times find occupation. But I cannot feel that human life and right should be held to be less sacred, nor that the labor of the physician should be no freer than that of the carpenter or blacksmith. I come to this great city, pass along its streets, and see all that modern progress has brought. I admire the marvellous development, and, while I admire it, as I should, I hope it will be a long time before we shall think of questioning the right of the ambulance to the right of way in our streets.

*Dr. W. W. Walker.*—We all like to hear an honest paper, coming from a truthful worker, and I believe that every word Dr. Howe has written and read to us this evening he honestly and truthfully believes. He has diagnosticated the case perfectly, but he has failed to give us a remedy. The remedy is a very simple one indeed. In the first place there are many essential things in the formation and the "running," as we may say, of a dental society, or any other organization. One of the most essential parts of any organization is its membership. When persons are admitted to a society they should attend religiously to the duties of that society, not simply have their names proposed, be admitted, and then stay away. It is very essential also that the finances of a society be carefully looked after if the society is to have success and influence, and there must also be harmony among the members. I contend that no organization, whether it be dental, medical, or any other, can be successful unless there is harmony among its members.

Dr. Howe spoke of the First District Dental Society, of which I was at one time quite an active member. He also mentioned the *Dental Cosmos.* Gentlemen, in working as an executive officer of a dental society, it will be found that there is much necessary data which cannot be obtained in the ordinary way or through every dental journal. There are not many good dental journals in the United States to-day, and I hold that among the first of them is the *Dental Cosmos.* From the publishers of the *Dental Cosmos* I

have received much information necessary to the proper carrying on of the business of the First District Dental Society; they have also given me notes that have been useful; but I do not believe they have ever paid for our printing. They have assisted us in a clerical way, and that assistance has been valuable, for they have given us the names and addresses of gentlemen in our profession that we could not get in any other way.

I repeat, Mr. President, that one of the most important factors in a dental organization is harmony. The very moment that we get rid of petty jealousies among members, just that moment can we bring out all the members to work in unison and harmony for the good of the society and of the dental profession. Then we will have found the only remedy for the abuses which have been spoken of. When the mass of dentists in a society, or in the city and State of New York, show a willingness to work for their society and for the dental profession, then the dental manufacturers and the dental journals will be compelled to come to us for support, and not until we have such harmony and unity of action among ourselves can we hope for it. Dentists must unite and work in earnest for their societies and their profession at large, instead of allowing personal interests and jealousies to control them. At this meeting of the Odontological Society, one of the best societies in the country, there is a very small representation of its members. It seems to show that other considerations than the welfare of the society and the profession govern their action; and it is so in the First District Society. Not until we are united and thoroughly in earnest can we fight our battles successfully and be independent of corporations; and when we are united they must come to us.

*Dr. Lord.*—Mr. President, while some one is getting ready to speak, I move that we give a very hearty vote of thanks to the authors of the very interesting and instructive papers that we have listened to this evening.

Motion carried.

*Dr. Bogue.*—Mr. President, I feel moved to make a word of thanks and acknowledgment to all the gentlemen who have enlightened us to-night. At the same time I want to call attention to a slight difference between my ideas and those expressed by the last speaker. What he says is so eminently true generally that I think it is well to call attention to one of the points he makes, which seems as though it were in opposition to the tenor of Dr. Howe's paper. He calls attention to, what no one knows better than he, the difficulty of running a dental society and keeping harmony within it. I have every reason to believe that his labors in that direction, the amount and extent of them, are known to very few persons, even though those efforts may accomplish great ends. I do not quite know if he meant to offer that as an excuse or not,—I do not think he did, but it was not clear,—as an excuse for our dependence more or less upon dental dealers. It seems to me that we have reached a point where we ought to take ground as a profession in favor of our own independence and freedom from the trammels of any company or organization whatever. It seems to me that the difficulty that attends the management of a dental society, which has been referred to, ought to incite us to a different kind of effort from any that we have put forth. For example, we find ourselves shut off from advertising such a little thing as I presented this evening. Nobody is to profit by it, and unless the dealers who publish the magazines can make a profit, they do not wish to publish any such appliance; for the reason that it can be made by every dentist in his own laboratory, and it is much more efficient than many expensive instruments. I was two years getting a little syringe made. The S. S. White Company at one time agreed to make it, but subsequently said they could not, and I finally had it made in Paris. I think it cost me sixty cents. It corresponds in every respect save one to the Dunn syringe, which costs, I believe, two dollars and fifty cents. The one respect in which it does not correspond to this syringe is that the contents of the Dunn syringe cannot be seen, but this drop-bottle syringe, being made of glass, shows exactly what is within. It is a cheaper and better instrument, but it is not procurable here.

Suppose we were to get from a pharmacist a formula for oxy-phosphate, or from a metallurgist a formula for an amalgam, and offer them for publication, how many publishers of existing dental journals would agree to publish them?

Quite lately I have been looking for a recipe which exists for soluble tooth powders. In the course of my researches I sent to the S. S. White Company for a box, and received one duly labelled "Soluble Tooth Powder;" I put some in a glass of water and tried for two weeks to dissolve it. As it did not dissolve in that time I thought it was not very soluble. The original recipe of that powder was one devised, I think, by J. D. White in his younger days. I suppose it is cheaper to use the label as it is and substitute some other materials than it would be to make it according to the original recipe. I do not know that that has been done, but after two weeks' trial to dissolve the soluble powder I was unsuccessful. If I can rediscover the formula and get it into the hands of my professional brethren it may be of benefit to them.

What I do not understand, and never have understood, is that we should so frequently endeavor to anticipate what the profit of the thing we are doing will be. That in a great measure will take care of itself. The questions for us are, how well can we accomplish our operation, with how little pain, how promptly, and how easily to use the things that our profession is so rich in ; the little devices that are so useful, and illustrations of which we do not often see. We all know that it is a little difficult to get such illustrations spread at large among the profession.

Our odontological friends in Great Britain have a superior magazine, and there is not an advertisement in it. The minutes of the meetings are published in a little pamphlet by themselves, and are so distributed to members. In that respect it is as professional as even our most severe critics could desire. Why should we not sufficiently emulate them as to have published in our interest the proceedings of our societies and the papers of our investigators and the theses of our students, and let the advertisements that may or may not come to those journals be dependent upon the wish of those advertising to place their wares before our twelve thousand dentists as a class? Why should it be necessary for us to pander to those advertisers sufficiently to urge them to advertise, and keep out of the columns of any such publication all matter that might in a sense be injurious to their trade interests? That is, as I understand it, the position that Dr. Howe has taken, and I feel grateful to him, and to others who have taken similar ground; and the moment that I see ideas of that kind spreading so that every man in my craft is anxious for the benefit, not only of the other members of the craft, but for the community at large who are served by us, then for all such we may cherish and express regard and respect. Moreover, it has been said that we are yet as a profession almost in our infancy; we have made some progress, but we cannot have the things we desire until we possess the means. Our course has been an open-handed one. We recognize the fact that we have received freely, and we should freely give without hope of reward. If we were all to take that high professional stand, it would not be long before the oculist, the aurist, the gynæcologist, and the dentist would all be recognized as being part and parcel of the healing art; and our materials could be purchased at a pharmacy, the same as with our friends of the other branches now. I hope, for one, that we will not be put in the attitude of fighting a trade-union. I support very heartily the body that will found its own institutions. I do not want to quarrel with the White Company, with the Welch Company, or any other company that furnishes good materials. If they furnish bad materials, I will quarrel with them. I should regret very much to see this society enter into a crusade, as against trade stores that are doing an honest business, and supplying good materials. On the other hand, I decidedly object to those dealers in materials, or the trade-unions, holding a hand over us, and consequently our receiving from them any advantages or compensation, or any tips, or what we may choose to call them.

*Dr. Dwinelle.*—I will say a single word, Mr. President, on this subject, rather than see it passed by. It seems to me that Dr. Howe's object is a very commendable one,—that is, to elevate our profession. It is true that we require not only the element of harmony, but we require every possible element in our progress that may tend to that great end. I do not think that the doctor has any intention of putting a firebrand in the community to incite it, or to divert it from its legitimate purposes. We have a code of ethics, recognized throughout the professional world, and it seems perfectly proper that we should be governed by it. I do not know why we should fail to be governed by those general ethical principles which govern the world at large, and have from the time civilization began, and yet make an exception in the case of dentistry. It seems to me that the doctor's paper has been entirely consistent with these ethical ideas, and I do not know why we should make an exception in our case. The element of harmony is desirable under all circumstances, but deference to this alone would not correct the mistake, or remedy the difficulty. We need something more, something higher.

In the medical profession, we all know that a man of intelligence will not take a remedy from his family physician, unless it is his privilege to know the constituents and the character of the ingredients the prescription is made of; and I see no reason why it should be different with the materials we use.

It seems to me that Dr. Howe's paper is a very proper one. That we are not perfect as a community of dentists is very true. We may make mistakes, but in due time we will profit by our experience and reach a higher status. It seems to me that this is one of the best papers I have ever listened to. It is one of the most important steps in the higher progress of our art and our profession, that I have had the privilege of becoming familiar with in a long time.

*Dr. J. F. P. Hodson.*—Mr. President, I rise to record my heartiest approval of the high general tone of the excellent paper to which we have been listening, and its cordial endorsement by the speakers who have preceded me argues that it has but reflected the good advance which has been made in the general mind of the profession in the direction of dignified professional status. Inasmuch, however, as special reference has been made in derogation of the fact of the clinics of the First District Dental Society being held in the establishment of the S. S. White Dental Manufacturing Company, it is but just that, as executive officer of the society, I point out that the clinics have been held precisely as now for very many years, and it can scarcely be questioned that their influence, and the good accomplished by them in all these years, has been very great. Their continuance is made mandatory upon the officers of the society, so they cannot be stopped without change in the by-laws, and lastly, change of place would necessitate a very large outlay for chairs and paraphernalia, and for the necessary room in which to place them and to hold the clinics. If the time has come for a change to be made, and if gentlemen really wish it, nothing is more easy than for them to attend the meetings of the society, and, by their propositions and votes, make that change, or any other.

*Dr. Dwinelle.*—I want to correct a mistake that Dr. Hodson has fallen into. It seems to me that what he refers to in his remarks did not have any special application to the paper to-night. The paper, I know, refers to these things as being unprofessional, and the question is, Is the statement correct? Is it unprofessional? If it is, then the argument has full force up to the present moment. If there is not money enough to overcome the difficulty in the matter of paying for a room elsewhere, and have the clinics held at some other place, that has nothing to do with the question. It is a misfortune that the society, of which I am a member and which I honor very much indeed, is unable to do in this matter what it would like to. It was founded by one of our dearest friends, and we should be jealous of its good name, and everything pertaining to its standing. I think Dr. Howe's proposition still stands true and uncontroverted,—that it is unprofessional for a society like ours to be under obligation to any foreign influence. We should have clean hands. If we are not able to furnish a room, that is our misfortune, but the proposition stands true all the same. We cannot afford to sacrifice principle to a beggarly contingency like this.

*Dr. Meriam.*—I think the position is something like this: It has been a question with many an English Mary, whether she should marry John, work hard, and be clothed in cheap apparel, or become the favorite of the squire's son, who would provide sumptuously, and deck her in silks and gay attire. And some, no doubt, have chosen that which seemed the easier way; but still, that is not the sort of prosperity we wish for those we love. Nor should we wish our profession to be supported by trade. Among the rules governing games at universities is one that those who play on the professional teams for hire cannot play on the university team. It seems to me that we could apply that rule very well in our profession. There is no dishonor in working for hire, and there is no added honor, perhaps, in working for a profession, but we cannot play for hire outside of the profession, and play inside of the profession at the same time.

*Dr. Dwinelle.*—We cannot serve God and Mammon.

*Dr. Meriam.*—That is it.

*Dr. Howe.*—Mr. President, I wish to say that no reference made in the paper has been for the purpose of criticising or fault-finding. Facts have been mentioned only to illustrate the object for which the paper was written. There seems to be, in the minds of some, considerable confusion, as is shown in various contributions to the discussion of this subject. There is no one who desires harmony more than I do, but there can be no harmony while such relations exist as must constantly suggest and provoke agitation and dissension. Lasting and real harmony can only rest on a basis of truth, and the profession, if it is a profession, must rest on a professional basis in order to have professional growth. I think we have no need so great at the present day as the cultivation of a professional feeling among dentists. Even the dissemination of knowledge, and the cultivation of desire for scientific inquiry, may be for the present considered secondary to the question, whether dentistry is to be really a profession, or to be hampered and bound by commercial control, so that it shall be able to do, and to use, only what shall prove advantageous to those who hold the power to dictate.

Adjourned.

S. E. Davenport, D.D.S., M.D.S.,
*Editor New York Odontological Society.*

